# A Power-Law Growth and Decay Model with Autocorrelation for Posting Data to Social Networking Services

**DOI:** 10.1371/journal.pone.0160592

**Published:** 2016-08-09

**Authors:** Toshifumi Fujiyama, Chihiro Matsui, Akimichi Takemura

**Affiliations:** 1 Department of Mathematical Informatics, Graduate School of Information Science and Technology, The University of Tokyo, Tokyo, Japan; 2 The Center for Data Science Edutation and Research, Shiga University, Shiga, Japan; Max-Planck-Institut fur Physik komplexer Systeme, GERMANY

## Abstract

We propose a power-law growth and decay model for posting data to social networking services before and after social events. We model the time series structure of deviations from the power-law growth and decay with a conditional Poisson autoregressive (AR) model. Online postings related to social events are described by five parameters in the power-law growth and decay model, each of which characterizes different aspects of interest in the event. We assess the validity of parameter estimates in terms of confidence intervals, and compare various submodels based on likelihoods and information criteria.

## Introduction

With the increasing use of social networking services (SNS), including blogs, Facebook, and Twitter, it is becoming increasingly important to extract information from SNS to optimize the use of resources for these events. Many models have been proposed to grasp people’s reactions to social events from various viewpoints, for instance, of complex networks [1] and time-series analyses [2, 3]. However, it is still difficult to qualitatively verify the models.

To overcome this problem, we use statistical methods in analyzing time-series data of social events. By assuming the power-law growth and decay for the number of postings about social events on SNS, we aim to gain insights into patterns of human interest regarding these events. We classify the patterns of human interest with parameters *α*: how “rapidly” people become interested/lose interest before/after an event, *β*: how “long” people remain interested in an event, and *γ*: how “much” attention is paid by people. The parameters, *α* and *β*, can be specified differently for data before and after the event. The last parameter *γ* only concerns the peak of the time series, and therefore, we consider a total of five parameters. The length of the time series of the data for social events is usually short, covering several weeks before and after the event. The time series are typically non-stationary because there is a sharp peak in the number of postings on the date of the event. Hence, we cannot use standard time series models, such as ARMA models [4].

Social events are usually scheduled in advance, and thus we call them predictable events as the time of occurrence is known in advance. On the other hand, there exist “unpredictable events” such as earthquakes, whose effect on the number of postings on SNS has been studied in [2, 5–7]. In the case of unpredictable events, we only obtain the data after the event. Whereas, for predictable events such as social events, we have the data before and after the event. Another type of event is a “deadline event” such as registration data collected within a deadline [8, 9]. The time series data for a deadline event often show similar behavior to unpredictable events. Since we deal with time series before and after the event separately using different parameters, our model consists of the growth part and the decay part, each of which is used to describe deadline and unpredictable events, respectively. For this reason, our method can be used for any of predictable, unpredictable, and deadline events.

The power-law distribution model is widely used, for instance, in time-series of daily views on YouTube [1] and rumor diffusion on Twitter [2]. The model was applied to blog posting about social events [3]. This model allows the prediction of the number of postings and the time to return to the normal activity level. Here we modify the model by introducing the conditional Poisson autoregressive (AR) model for deviations of data from the power-law model. The conditional Poisson AR model is often used in fields of social science such as economics, political science, and epidemiology [10, 11]. The theoretical aspects of the conditional Poisson AR model are found in [12–14]. Thus, we propose the model consisting of the power-law growth and decay model and the conditional Poisson AR model. The former describes the expected number of postings, while the latter describes deviations from the power-law growth and decay model. We give interpretations of the parameters contained in the model. We show that we are able to obtain the necessary information from the parameter values. The advantage of employing a statistical model is that we can assess the validity of parameter estimates in terms of confidence intervals and we can compare various submodels based on likelihoods and information criteria.

The organization of this paper is as follows. After describing the data, we first introduce the five-parameter power-law growth and decay model with independent Poisson distributions. Then the model is extended to the one combined with the conditional Poisson AR model by introducing autocorrelation. We then analyze the data using these models. This paper ends up with concluding remarks.

## Materials and Methods

### Data description

The data analyzed in this study is provided by NTTCom Online Marketing Solutions Corporation through the BuzzFinder service (Those who want to access the data we used can request it through the web page http://www.nttcoms.com/contact/). We have used two millions of blog postings collected over six months, from January 2014 to June 2014. From more than ten popular Japanese blog-servers, including goo-blog, ameblo, yahoo blog, and livedoor blog, blog postings are counted, if they contain a keyword we specified.

**Fig 1:**
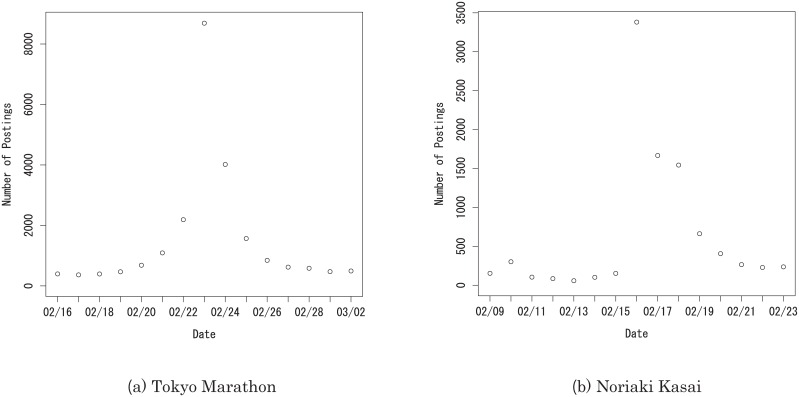
Number of postings in Japanese about (a) 2014 Tokyo Marathon on BuzzFinder and (b) Noriaki Kasai during the 2014 Winter Olympics. (a) shows the typical symmetric pattern, while (b) shows the strong asymmetric pattern.

In Fig 1(a) we show a typical symmetric pattern for the number of postings. The Tokyo Marathon 2014 was held on Sunday, February 23, 2014. It was highly anticipated and it ended without anything unexpected happening. In this and similar events, the number of postings shows a symmetric pattern with a sharp peak on the day of the event. Both sides of the peak seem to exhibit negative power behavior in the time difference from the actual date of the event.

An asymmetric pattern is observed when there is a surprising element to the event. In Fig 1(b) we show the number of postings on Noriaki Kasai regarding February 16, 2014, when he won a silver medal in the ski jump at the 2014 winter Olympics. From the data we see that people did not anticipate the medal before the event.

In view of the above characteristics of the number of postings, we propose that SNS posting data presents an interesting challenge for statistical analysis. Since the data is in a time series, we propose models that can account for autocorrelations. As we show in the Results section, our proposed model fits the data very well.

### A power-law growth and decay model for the mean number of postings

We propose a model without autocorrelation and an autoregressive model. We then compare these models by Akaike’s Information Criterion (AIC) in the Results section.

#### The power-law growth and decay model without autocorrelation

Let *t*_0_ denote the date of the event and let *y*_*t*_ denote the number of posts on day *t* about the event. We model the expected value of *y*_*t*_ by the following power-law growth and decay function:

$$\begin{array}{c} E\left(y_{t}\right)=\mu_{t}\left(\alpha_{b},\alpha_{a};\beta_{b},\beta_{a};\gamma \right)=\left\{\begin{array}{c} \gamma \frac{1}{{\left(\alpha_{b}\left(t_{0}-t\right)+1\right)}^{\beta_{b}}}\;\text{for}\;t<t_{0}\\ \gamma \frac{1}{{\left(\alpha_{a}\left(t-t_{0}\right)+1\right)}^{\beta_{a}}}\;\text{for}\;t>t_{0}. \end{array}\right. \end{array}$$ (1)

*γ* is a common parameter for *t* < *t*_0_ and *t* > *t*_0_. The power-law growth and decay model was proposed for the symmetric case in [3] without the parameter *α*. They used the least-squares method, while we use the maximum likelihood method for parameter estimation. They do not consider fitting the data close to the peak, which we do by introducing the parameter *α*. Note that the model (1) consists of the growth part (*t* < *t*_0_) and the decay part (*t* > *t*_0_), each of which models deadline events and unpredictable events, respectively.

The interpretation of the parameters is as follows.

*α*_*a*/*b*_ steepness of the curve just before/after the event. How rapidly an event gains/loses interest.
*β*_*a*/*b*_ longer growth/decay pattern. How long people get/remain interested in an event.
*γ* impact of the event (peak level, the maximum number of postings). The total attention paid to an event.

When *α*_*a*_ = *α*_*b*_ and *β*_*a*_ = *β*_*b*_, the model (1) shows the symmetric pattern. For the symmetric case, we assume that *y*_*t*_, *t* = *t*_*L*_, *t*_*L*_ + 1, …, *t*_*U*_ − 1, *t*_*U*_, *t*_*L*_ ≤ *t*_0_ ≤ *t*_*U*_, are independent Poisson random variables with mean *μ*_*t*_(*α*, *β*, *γ*) in Eq (1). We call this the power-law growth and decay model without autocorrelation. We denote the probability function for the Poisson distribution with mean *μ* as

$$\begin{array}{c} \text{Po}\left(y|\mu \right)=\frac{\mu^{y}}{y!}e^{-\mu }. \end{array}$$ (2)

Then the likelihood function is

$$\begin{array}{c} L(\alpha ,\beta ,\gamma )=\prod_{t=t_{L}}^{t_{U}}\text{Po}\left(y_{t}|\mu_{t}\left(\alpha ,\beta ,\gamma \right)\right)=\prod_{t=t_{L}}^{t_{U}}\frac{\mu_{t}{\left(\alpha ,\beta ,\gamma \right)}^{y_{t}}}{y_{t}!}e^{-\mu_{t}\left(\alpha ,\beta ,\gamma \right)}. \end{array}$$ (3)

**Fig 2:**
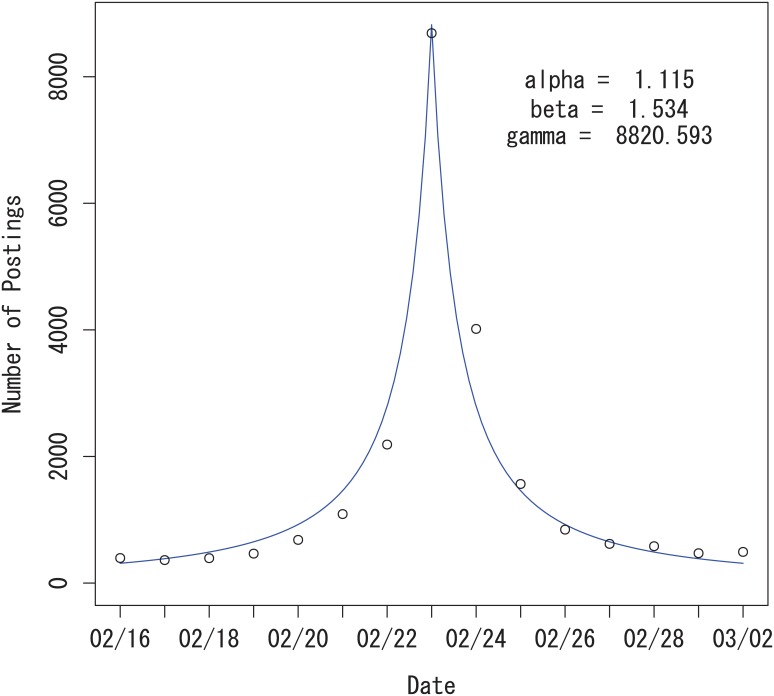
Power-law growth and decay model without autocorrelation for the Tokyo Marathon data. The solid line is the observed data and the dotted line is obtained from the power-law growth and decay model.

Maximization of the log-likelihood function is numerically straightforward. We fitted a Poisson distribution to the Tokyo Marathon data in Fig 2. We chose the estimation period as one week before (*t*_*L*_ = *t*_0_ − 7) and after (*t*_*U*_ = *t*_0_ + 7) the event for the reason discussed later in the Results section. The parameter estimates are $\hat{\alpha }=1.115$, $\hat{\beta }=1.534$ and $\hat{\gamma }=8820.593$. Our model seems to fit the data well, but there is a slight asymmetry in this data, which is not captured by the symmetric model in Eq (1).

#### Conditional Poisson regression model for autocorrelations

In the power-law growth and decay model without autocorrelation we assumed that the number of postings *y*_*t*_ are independent. We generalize this model to allow autocorrelations by using a conditional Poisson regression model. As we saw, the estimate for the parameter *γ* in Eq (1) is very close to *y*_*t*_0__. Hence, in this section we replace *γ* by *y*_*t*_0__ and consider the conditional likelihood given *y*_*t*_0__. This is the initial value of our autoregressive scheme and we model the number of postings after the event *y*_*t*_, *t* > *t*_0_. Conditional likelihoods are much simpler than unconditional likelihoods and are often used in statistical time series analyses [15].

Considering the data *y*_*t*_, *t* < *t*_0_, before the date of the event, we propose to use the model given in Eq (4) by reversing the time axis. This is similar to the standard AR(1) process *x*_*t*_ = *ρx*_*t* − 1_ + *ϵ*_*t*_, where the reciprocal of the autoregressive coefficient *ρ* is used. However this model for the data preceding the event is somewhat unsatisfactory, in particular for the purpose of predicting *y*_*t*_0__ before the date of the event. We discuss this point again in the Discussion section.

We let *t*_0_ = 0 for simplicity and replace *γ* by *y*_0_ in Eq (1) and take as the conditional expected value of *y*_*t*_ given *y*_0_

$$\begin{array}{c} E\left(y_{t}|y_{0}\right)=y_{0}\frac{1}{{\left(\alpha t+1\right)}^{\beta }},\;t\geq 0. \end{array}$$

Then *E*(*y*_*t*_|*y*_0_) is written recursively as

$$\begin{array}{ll} E(y_{t}|y_{0}) & =E(y_{t-1}|y_{0}){\left(\frac{\left(t-1\right)\alpha +1}{t\alpha +1}\right)}^{\beta }\\ & =E(y_{t-2}|y_{0}){\left(\frac{\left(t-2\right)\alpha +1}{t\alpha +1}\right)}^{\beta }\\ & =\cdots \end{array}$$

We propose the following AR(2) model for *y*_*t*_, *t* ≥ 2:

$$\begin{array}{lll} p(y_{1}|y_{0}) & = & \text{Po}(y_{1}|y_{0}\frac{1}{{\left(\alpha +1\right)}^{\beta }})\\ p(y_{2}|y_{1},y_{0}) & = & \text{Po}(y_{2}|w\times y_{1}{\left(\frac{\alpha +1}{2\alpha +1}\right)}^{\beta }+\left(1-w\right)\times y_{0}{\left(\frac{1}{2\alpha +1}\right)}^{\beta })\\ & & \vdots \\ p(y_{m}|y_{m-1},y_{m-2}) & = & \text{Po}\left(y_{m}|w\times y_{m-1}{\left(\frac{\left(m-1\right)\alpha +1}{m\alpha +1}\right)}^{\beta }+\left(1-w\right)\times y_{m-2}{\left(\frac{\left(m-2\right)\alpha +1}{m\alpha +1}\right)}^{\beta }\right). \end{array}$$ (4)

Note that *y*_1_ is given in an AR(1) form. The parameter *w* linearly connects the AR(1) model and the complete AR(2) model, which is solely determined from the information two days ago. Therefore, the containing ratio of the AR(1) model in Eq (2) is given by this parameter.

Then the conditional likelihood function for *α*, *β*, *w*, given *y*_0_, for the data *y*_1_, …, *y*_*T*_ is

$$\begin{array}{c} L\left(\alpha ,\beta ,s\right)=\text{Po}\left(y_{1}|y_{0}\frac{1}{{\left(\alpha +1\right)}^{\beta }}\right)\\ \times \prod_{m=2}^{T}\text{Po}\left(y_{m}|w\times y_{m-1}{\left(\frac{(m-1)\alpha +1}{m\alpha +1}\right)}^{\beta }+\left(1-w\right)\times y_{m-2}{\left(\frac{(m-2)\alpha +1}{m\alpha +1}\right)}^{\beta }\right). \end{array}$$ (5)

When we estimate *w* in *L*(*α*, *β*, *w*), we restrict *w* ∈ [0, 1], although unrestricted MLE of *w* may result in *w* > 1.

Note that the model without autocorrelation in Eq (3) and the AR(2) model in Eq (5) are separate models. In the usual AR(1) model for continuous observations *x*_*t*_ = *ρx*_*t* − 1_ + *ϵ*_*t*_, the model without autocorrelation is a special case of *ρ* = 0. In order to interpolate between the model without autocorrelation Eq (3) and the AR(2) model (5), we also propose the following unified model with additional parameters *u*, *v* ∈ [0, 1] representing the weights of the two models:

$$\begin{array}{ll} p(y_{m}|y_{m-1},y_{m-2}) & =\text{Po}(y_{m}|w\times {\left(\frac{y_{0}}{{\left(\left(m-1\right)\alpha +1\right)}^{\beta }}\right)}^{u}\times y_{m-1}^{1-u}{\left(\frac{\left(m-1\right)\alpha +1}{m\alpha +1}\right)}^{\beta }\\ & +\left(1-w\right){\left(\frac{y_{0}}{{\left(\left(m-2\right)\alpha +1\right)}^{\beta }}\right)}^{v}\times y_{m-2}^{1-v}{\left(\frac{\left(m-2\right)\alpha +1}{m\alpha +1}\right)}^{\beta }). \end{array}$$ (6)

This unified model reduces to the model without autocorrelation for *u* = *v* = 1, while it results in the AR(2) model for *u* = *v* = 0. We introduced the parameters *u* and *v* separately for increased flexibility.

## Results

### Analysis of Japanese social networking data

We apply our models to SNS data in Japan. The data is summarized in Table 1. In Table 1, “Date” is the date of the event in the format month/day in 2014. “ID” is our identifier for the events used in later tables. “Searchword” is the word we used in the BuzzFinder service to search for the postings related to the events. “Remarks” are the explanations of the events. The searchwords we have used are related to national holidays, major sports events, and cultural events held in the first half of 2014, in order to collect enough amount of data for statistical analysis.

**Table 1:** List of events that took place in 2014, and corresponding search terms which were considered in this study.

Date	ID	Searchwords	Remarks
1/13	Seijin	Seijin no Hi	Coming-of-Age Day
1/26	O-Marathon	Osaka International Ladies Marathon	
2/3	Setsubun	Setsubun	Bean Throwing Festival
2/8	Sochi	Sochi Olympics	The Opening Ceremony
2/9	Uemura	Aiko Uemura	Ladies’ Moguls Final
2/11	Kenkoku	Kenkokukinenbi	National Foundation Day
2/14	Valentine	Valentine’s Day	
2/15	Hanyu	Yuzuru Hanyu	Men’s Singles Free Skating
2/16	Kasai	Noriaki Kasai	Men’s Large Hill Final
2/21	Asada	Mao Asada	Ladies’ Singles Free Skating
2/23	T-Marathon	Tokyo Marathon	
3/3	Academy	Academy Awards	Oscar Ceremony
3/11	Earthquake	Great East Japan Earthquake	
3/14	White	White Day	
3/21	Shunbun	Shunbun no Hi	Vernal Equinox Day
3/24	mayoral	Osaka Mayoral Elections	
5/5	Kodomo	Kodomo no Hi	Children’s Day
6/9	Oshima	Yuko Oshima	AKB(Japanese idol girl group)’s oncert
6/15	W-cup	The World Cup	Japan vs. Cote d’Ivoire
6/21	Geshi	Geshi	Summer Solstice

#### Parameter estimation for the power-law growth and decay model without autocorrelation

For the model without autocorrelation, we chose the estimation period as one week before (*t*_*L*_ = *t*_0_ − 7) and after (*t*_*U*_ = *t*_0_ + 7) the event. In Tables 2 and 3 we show parameter estimation of the model without autocorrelation fitted to the SNS data collected over 4, 7, 14, and 21 days before and after the events. As the estimation period becomes longer, the parameter *α* tends to be larger, while *β* tends to be smaller by converging to 1. The parameter estimation over a long estimation period seems to be affected by a small number of postings far from the issue of an event. On the other hand, the parameter values estimated for a short estimation period seem to be strongly affected by the peak. For the best reflection of the behavior around the peak, we have chosen the estimation period as one week before and after the event.

**Table 2:** Parameter estimates for the power-law growth and decay model without autocorrelation, fitted to the number of postings in Japanese about TokyoMarathon, 2014.

Estimation period	*α*_*b*_	*β*_*b*_	*γ*	*α*_*a*_	*β*_*a*_
4	0.518	2.556	8733.476	0.172	5.388
7	1.115	1.534	8820.593	0.635	1.932
14	1.416	1.348	8860.41	0.812	1.66
21	1.835	1.191	8905.306	1.001	1.479

**Table 3:** Parameter estimates for the power-law growth and decay model without autocorrelation, fitted to the number of postings in Japanese about Valentine’s day, 2014.

Estimation period	*α*_*b*_	*β*_*b*_	*γ*	*α*_*a*_	*β*_*a*_
4	1.295	1.488	55659.58	0.597	2.318
7	1.545	1.347	55765.31	0.748	1.986
14	2.306	1.106	56042.61	1	1.668
21	2.799	1.018	56171.68	1.167	1.536

In Table 4 we show parameter estimation of the model without autocorrelation fitted to the SNS data collected over the estimation period one week before and after the events. Because the distribution of postings about events often showed asymmetry, we estimated the before-event parameters *α*_*b*_, *β*_*b*_ and the peak level *γ* for one week before the event, and then estimated the after-event parameters *α*_*a*_, *β*_*a*_ separately with the same *γ* as the before-event parameters. We also computed 95% confidence intervals based on the Fisher information matrix (S1) and the asymptotic normal approximation of the sampling distribution of parameter estimates.

**Table 4:** Parameter estimates for the power-law growth and decay model without autocorrelation.

ID	*α*_*b*_	*β*_*b*_	*γ*	*α*_*a*_	*β*_*a*_
Seijin	0.889 (0.029)	1.493 (0.022)	53660 (585)	0.908 (0.022)	1.463 (0.019)
O-Marathon	7.902 (2.48)	0.858 (0.082)	993 (31.5)	0.207 (0.044)	4.337 (0.718)
Setsubun	2.694 (0.055)	1.318 (0.012)	105334 (138)	0.397 (0.007)	2.921 (0.035)
Sochi	0.179 (0.02)	5.499 (0.479)	5479 (72.6)	1.089 (0.086)	1.65 (0.069)
Uemura	0.346 (0.057)	3.256 (0.363)	4992 (77.0)	0.231 (0.023)	3.436 (0.258)
Kenkoku	1.405 (0.101)	2.012 (0.075)	7708 (89.6)	1.021 (0.068)	2.122 (0.079)
Valentine	3.498 (0.018)	0.951 (0.011)	55539 (413)	0.742 (0.018)	1.991 (0.028)
Hanyu	0.898 (0.043)	1.823 (0.048)	10317 (96.8)	2.353 (0.158)	0.891 (0.025)
Kasai	599800.772 (4200)	0.221 (0.003)	3368 (57.9)	0.131 (0.021)	4.584 (0.593)
Asada	0.447 (0.018)	2.755 (0.073)	18898 (139)	1.289 (0.061)	1.047 (0.024)
T-Marathon	2.327 (0.169)	1.186 (0.036)	8702 (94.4)	0.619 (0.036)	1.948 (0.068)
Academy	39.489 (13.7)	0.423 (0.03)	2923 (54.1)	3.97 (0.85)	0.302 (0.022)
Earthquake	10.695 (0.607)	0.822 (0.013)	41580 (193)	2.611 (0.087)	1.185 (0.017)
White	6.285 (0.279)	0.691 (0.009)	45490 (298)	0.309 (0.01)	2.483 (0.054)
Shunbun	2.99 (0.185)	1.259 (0.031)	14380 (116)	0.902 (0.045)	1.969 (0.056)
Mayoral	0.096 (0.007)	7.803 (0.47)	1632 (37.9)	1.217 (0.178)	1.375 (0.102)
Kodomo	14.37 (1.12)	0.783 (0.016)	29200 (154)	0.637 (0.021)	2.316 (0.047)
Oshima	1.083 (0.109)	1.136 (0.055)	4456 (66.7)	0.673 (0.06)	1.23 (0.062)
W-cup	0.013 (3.61 × 10^−4^)	34.707 (0.96)	45930 (157)	1.091 (0.038)	0.787 (0.014)
Geshi	8.096 (0.893)	0.746 (0.025)	8183 (90.1)	0.766 (0.047)	1.498 (0.052)

**Fig 3:**
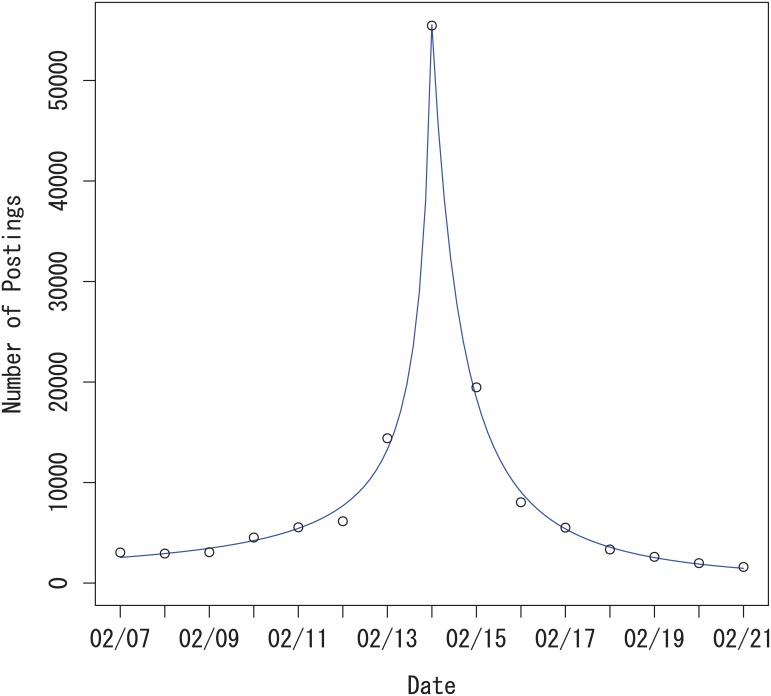
Valentine’s Day 2014 power-law growth and decay model without autocorrelation. Interpretation of the lines is the same as in Fig 2.

In Fig 3 we show the data for postings about Valentine’s day, around February 14, 2014. The graph looks almost symmetric, but the estimated before-event parameters and after-event parameters are different. In Fig 3 the slope just before the date of the event is steeper than after the event and the number of postings decreases to zero faster after the event than before the event. The estimated parameters in Table 5 reflect this behavior.

**Table 5:** Parameter estimates for the power-law growth and decay model without autocorrelation, fitted to the number of postings in Japanese about Valentine’s day, 2014.

log-likelihood	95% Confidence interval	Standard error
*α*_*b*_ = 3.498	3.267 < *α*_*b*_ < 3.728	0.118
*β*_*b*_ = 0.951	0.929 < *β*_*b*_ < 0.973	0.011
*γ* = 55539	54728 < *γ* < 56349	413
*α*_*a*_ = 0.742	0.707 < *α*_*a*_ < 0.777	0.018
*β*_*a*_ = 1.991	1.935 < *β*_*a*_ < 2.046	0.028

The parameter estimates for the power-law growth and decay model without autocorrelation fitted to postings related to a number of different events are given in Table 4. The standard errors of the estimates are shown in parentheses. We see that the parameter estimates are generally reliable, except for the data labeled by ID: Kasai and ID: W-cup, since the standard errors are relatively small compared to the estimates. Some events show strongly asymmetric patterns, which are reflected in the large differences between the before-event and after-event parameter estimates.

The disagreement of the model with Kasai’s data lies in the nature of the data. Kasai’s data shows steep growth for *t* < *t*_0_ (Fig 1(b)), which implies that the event belongs to the unpredictable type. For this reason, the growth part of the model does not fit the data, although the decay part fits the data well. On the other hand, the model disagrees with W-cup’s data because of another reason. According to the collected data of W-cup, there are several peaks in a short interval. It is then understood that the poor fit of out model to W-cup’s data is due to the existence of another peak before the parameter *β* converges. This implies the limitation of adaptation of our model, that is, the power-law growth and decay model only fits single-peak data.

Based on the power-law growth and decay model without autocorrelation, we considered predicting the after-event parameters based on the data before the event. However, this was difficult because of the asymmetry of many events. To explain this phenomenon, we performed a multiple regression analysis, where the before-event parameters *α*_*b*_, *β*_*b*_, *γ* are explanatory variables and the after-event parameters *α*_*a*_, *β*_*a*_ are objective variables, but we did not find a significant correlation.

#### Parameter estimation for the AR(2) model

For the AR(2) model, we chose the estimation period as two week after (*t*_*U*_ = *t*_0_ + 14) the event. In Tables 6 and 7 we show the parameter estimation of the AR(2) model fitted to the SNS data as 4, 7, 14, and 21 days before and after the events. As the estimation period becomes longer, the estimation of the parameters *α*, *β*, and *s* becomes stable. We have chosen the estimation period as two weeks after the event, which seems to be long enough for the parameter estimation to be stable from Tables 6 and 7.

**Table 6:** Parameter estimates for AR(2) model, fitted to the number of postings in Japanese about TokyoMarathon, 2014.

Estimation period	*α*	*β*	*s*
4	0.165	5.582	0.409
7	0.331	2.934	1
14	0.352	2.788	1
21	0.364	2.711	1

**Table 7:** Parameter estimates for AR(2) model, fitted to the number of postings in Japanese about Valentine’s day, 2014.

Estimation period	*α*	*β*	*s*
4	0.685	2.18	0.009
7	0.722	2.04	0.057
14	0.784	1.924	0.244
21	0.789	1.913	0.303

**Fig 4:**
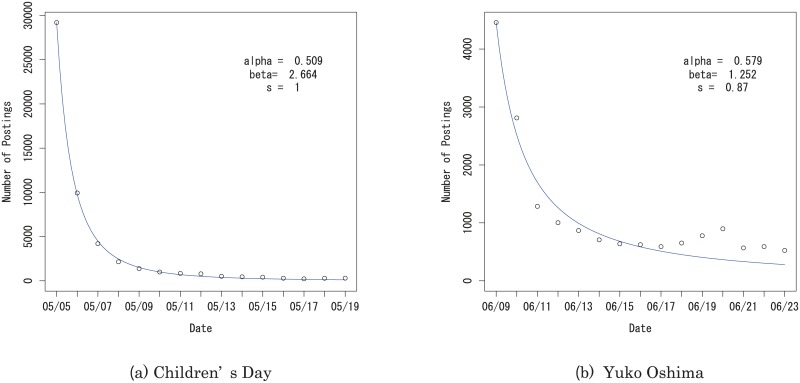
Fits of the AR(2) model to the number of postings about (a) Children’s Day and (b) Yuko Oshima.

In Table 8 we show the fit of the AR(2) model to our data. In Table 8 “log-lik.” stands for the log-likelihood for the estimated model. Fig 4 shows the fit of the AR(2) model for “Children’s Day (Japan) 2014” and for “Yuko Oshima (Japanese actress)” as representative examples. The parameter *s* is estimated as *s* = 1 for “Children’s Day” (Fig 4(a)), it is estimated as *s* = 0.87 for “Yuko Oshima” (Fig 4(b)). The parameter *s* reflects the longevity of interest in the event. The parameter *s* tends to be close to 1 for events with faster decay rates, but tends to be less than one for events with long-lasting interest. This is reasonable, because 1 − *s* represents the effect of two days before and *s* = 1 means that the autocorrelation is fully explained only by the number of postings one day before. We compared the AICs for the AR(2) model with *s* = 1 and *s* ≠ 1. For many data sets, the AIC was smaller when *s* was estimated to be less than 1.

**Table 8:** Parameter estimation for the AR(2) model.

	AR(2) model						*s* = 1	
ID	*α*	*β*	*γ*	*s*	log-lik.	AIC	log-lik.	AIC
Seijin	0.691	1.675	53188	1.000	-585.919	1177.838	-585.919	1175.838
O-Marathon	0.147	5.605	992	0.697	-79.524	165.048	-81.073	166.145
Setsubun	0.353	2.946	105410	1.000	-4034.221	8074.441	-4034.218	8072.436
Sochi	1.045	1.654	5374	0.797	-99.805	205.61	-100.837	205.674
Uemura	0.37	2.447	5348	0.784	-238.48	482.959	-243.033	490.065
Kenkoku	0.705	2.67	7679	1.000	-88.261	182.522	-88.261	180.521
Valentine	0.784	1.924	55456	0.244	-288.888	583.776	-395.427	794.854
Hanyu	2.122	0.896	9969	1.000	-224.275	454.549	-224.275	452.549
Kasai	0.265	2.457	3381	0.698	-462.99	931.98	-480.557	965.113
Asada	1.236	1.093	18939	0.636	-233.129	472.257	-252.261	508.521
T-Marathon	0.352	2.788	8688	1.000	-239.606	485.211	-239.606	483.221
Academy	0.621	0.807	2923	1.000	-1031.521	2069.041	-1031.52	2067.041
Earthquake	2.907	1.142	41566	0.125	-426.22	858.439	-822.486	1648.972
White	0.381	2.132	45438	0.248	-441.324	888.648	-618.75	1241.499
Kodomo	0.509	2.664	29201	1.000	-179.851	365.702	-179.851	363.702
Oshima	0.579	1.252	4458	0.87	-320.426	646.853	-323.49	650.98

#### Parameter estimation for the unified model

In Table 9, we apply the unified model (6) to the data. In Table 10 we compare the unified model and some relevant AR(1) models (*w* = 1) based on the AIC. The leftmost column shows the AICs of the unified AR(2) model and the second leftmost column gives the AICs of the unified AR(1) model. The third and fourth column show the AICs of the unified AR(1) model at the extreme values *u* = 1, 0. For some cases the unified AR(1) model provides the smallest AIC, even at the extreme values. This suggests that the most general model considered here (the unified AR(2) model) is over-parameterized for some events and the maximum likelihood estimation is not very stable for these cases.

**Table 9:** Parameter estimates for the unified model.

ID	*α*	*β*	*γ*	*w*	*u*	*v*	log-lik.	AIC
Seijin	0.69	1.676	53188	1	0	1	-585.926	1181.851
O-Marathon	0.282	3.33	992	0.986	0.757	0.99	-74.444	158.889
Setsubun	0.354	2.944	105410	1	0	1	-4034.298	8078.596
Sochi	1.239	1.472	5374	0.849	0.103	0.927	-100.595	211.19
Uemura	0.326	2.647	5348	1	0	1	-243.033	496.065
Kenkoku	0.705	2.67	7679	1	0	0	-88.261	186.522
Valentine	0.807	1.893	55456	0	1	0	-302.381	614.762
Hanyu	2.527	0.809	9969	0.841	0	1	-217.695	445.39
Kasai	0.447	1.756	3381	0.121	0	1	-340.758	691.516
Asada	1.091	1.179	18939	0.718	0.14	0.115	-234.296	478.593
T-Marathon	0.529	2.044	8688	0.25	0.25	0.93	-237.996	485.991
Academy	0.353	1.077	2923	0.372	0	1	-751.117	1512.235
Earthquake	2.993	1.13	41566	0	1	0	-435.54	881.08
White	0.443	1.904	45438	0.329	0.912	0.12	-444.672	889.344
Kodomo	0.509	2.665	29201	1	0	0	-179.852	369.704
Oshima	0.9	0.895	4458	0.968	0.257	0.501	-310.51	631.019

**Table 10:** Comparison of the power-law growth and decay and unified models based on the AIC.

		*w* = 1			
				*u* = 1	*u* = 0
ID	AIC	*u*	AIC	AIC	AIC
Seijin	1181.851	0	1177.838	1842.099	1175.838
O-Marathon	158.889	0.76	154.885	154.618	166.145
Setsubun	8078.596	0	8074.437	15358.336	8072.436
Sochi	211.19	0.217	207.276	237.042	205.674
Uemura	496.065	0	492.065	694.523	490.065
Kenkoku	186.522	0	182.522	193.093	180.521
Valentaine	614.762	0.845	718.707	721.387	794.854
Hanyu	445.39	0.134	442.973	788.555	452.549
Kasai	691.516	0.824	681.578	691.943	965.113
Asada	478.593	0.332	480.051	586.74	508.521
T-Marathon	485.991	0.464	478.231	505.845	483.221
Academy	1512.235	0.634	1542.979	1714.677	2067.041
Earthquake	881.08	1	1121.295	1119.295	1648.972
White	899.344	0.864	1024.115	1029.941	1241.499
Kodomo	369.704	0	365.703	464.977	363.702
Oshima	631.019	0.269	626.589	799.469	650.98

## Discussion and Conclusion

In this paper, we proposed a power-law growth and decay model combined with a conditional Poisson AR model. The conditional Poisson AR model was introduced to model deviations from the power-law growth and decay model. The power-law growth and decay model contained five parameters, which determined how rapidly interest in an event grew, how long people remained interested in an event, and how much attention was paid. The first two contribute four parameters, since the parameters for before and after an event can be modeled separately. Also we compared the models based on AIC systematically in Tables 8, 9 and 10.

In spite of good fits of our model, a number of issues remain. Since the lengths of the datasets considered are fairly short, the unified model in Eq (6) with five parameters is probably over-parameterized. This was reflected in the AIC values which showed that models with fewer parameters provided a better fit to the data.

Although we assumed a single peak at *t* = *t*_0_ in the data, some events, such as the Olympic games, may admit more peaks in postings due to their long duration. The number of postings during an event with a longer duration usually reveals a more complicated pattern. The patterns at the beginning and the end of the event seem to be similar to those for single-day events, whereas the pattern around the day of the event is noticeably different. It is not clear how to generalize our model for events with a longer duration.

Furthermore, in order to predict patterns in social networking data, we need to know the peak level *γ* for the number of postings and the after-event parameters *α*_*a*_ and *β*_*a*_ from the shape of the before-event pattern in advance. The estimation of these parameters is difficult for our data, which suggests that the before- and after-event parameters are independent. For this reason, we estimated the before- and after-event parameters separately, although this is somewhat unsatisfactory for our purposes. Our conditional Poisson AR model is not suitable for predictive purposes. In addition, the prediction of unusual patterns in the post-event data is difficult for certain types of events. To improve the predictive capability of the model, we could include characteristics of the event in the model. For instance, for events set on fixed dates, such as national holidays, we can analyze the inter-annual stability of patterns.

## Supporting Information

S1 File Fisher information matrix for the model without autocorrelation. We presented the Fisher information matrix for the model without autocorrelation, which is needed for the construction of confidence intervals for parameter estimates in Fig 3 and Table 4.
(PDF)
