# Supplementary material for: A Power-Law Growth and Decay Model with Autocorrelation for Posting Data to Social Networking Services
Source: PLoS One. 2016 Aug 9;11(8):e0160592. doi: 10.1371/journal.pone.0160592 (PMC4978406; doi:10.1371/journal.pone.0160592)
Supplement: S1 File — We presented the Fisher information matrix for the model without autocorrelation, which is needed for the construction of confidence intervals for parameter estimates in Fig 3 and Table 4. (PDF) [file pone.0160592.s001.pdf]

A power-law growth and decay model with autocorrelation for posting data to social networking services

Toshifumi Fujiyama[1*], Chihiro Matsui[1*], Akimichi Takemura[1],

1 Department of Mathematical Informatics, Graduate School of Information Science and Technology, The University of Tokyo, Tokyo, Japan

*** tosi.fujiyama@gmail.com (TF); matsui@mist.i.u-tokyo.ac.jp (CM)**

Supporting Information

Fisher information matrix for the model without autocorrelation In appendix we present the Fisher information matrix for the model without autocorrelation. This is needed for the construction of confidence intervals for the parameter estimates in Fig 3 and Table 4.

As noted, the time series considered here are short, and the data is non-stationary. Hence, the usual asymptotics for the length of the time series are not appropriate. Nevertheless it is of theoretical interest to consider the behavior of the MLE of (3) as the length of the series increases. On the other hand we have a large number of postings y_{t_0} on the date t_0 of the event. Under our Poisson model, we can also consider the asymptotics, where $\gamma = E(y_{t_0})$ tends to infinity. We calculate the Fisher information matrix as follows,

$$I = \begin{pmatrix} I_{\alpha\alpha} & I_{\alpha\beta} & I_{\alpha\gamma} \\ I_{\alpha\beta} & I_{\beta\beta} & I_{\beta\gamma} \\ I_{\alpha\gamma} & I_{\beta\gamma} & I_{\gamma\gamma} \end{pmatrix},$$

to gain insight into the behavior of the MLE for the model (3).

For notational simplicity let $t_0 = 0$ and $t_L \le 0 \le t_U$. Then the log-likelihood function $l(\alpha,\beta,\gamma) = \log L(\alpha,\beta,\gamma)$ is written as

$$\begin{aligned} l(\alpha,\beta,\gamma) &= \sum_{t=t_L}^{t_U} (y_t \log \mu_t(\alpha,\beta,\gamma) - \mu_t(\alpha,\beta,\gamma) - \log y_t!) \\ &= \sum_{t=t_L}^{t_U} \Big(y_t(\log\gamma - \beta\log(\alpha|t|+1)) - \gamma\frac{1}{(\alpha|t|+1)^\beta} - \log y_t! \Big) \\ &= C + \log\gamma \sum_{t=t_L}^{t_U} y_t - \beta \sum_{t=t_L}^{t_U} y_t \log(\alpha|t|+1) - \gamma \sum_{t=t_L}^{t_U} \frac{1}{(\alpha|t|+1)^\beta}, \end{aligned}$$

where C is a constant. Then

$$-\frac{\partial^2}{\partial\alpha^2} l = -\beta \sum_{t=t_L}^{t_U} y_t \frac{|t|^2}{(\alpha|t|+1)^2} + \gamma\beta(\beta+1) \sum_{t=t_L}^{t_U} \frac{|t|^2}{(\alpha|t|+1)^{\beta+2}}.$$

The expected value of the second derivative is

$$I_{\alpha\alpha} = E\Big(- \frac{\partial^2}{\partial \alpha^2} l \Big) = \gamma \beta^2 \sum_{t=t_L}^{t_U} \frac{|t|^2}{(\alpha|t|+1)^{\beta+2}}.$$

Note that $I_{\alpha\alpha} \to \infty$ as $\gamma = E(y_0) \to \infty$. However, when γ is fixed

$$\lim_{\max(-t_L, t_U) \to \infty} I_{\alpha\alpha} = \infty \quad \Leftrightarrow \quad \beta \leq 1 \quad \Leftrightarrow \quad \sum_{t=t_L}^{t_U} E\left(y_t\right) \to \infty.$$

To obtain an expression for $I_{\alpha\beta}$, we first note that

$$(\alpha t + 1)^{-\beta} = \exp(-\beta \log(\alpha t + 1))$$

and

$$\frac{\partial}{\partial \beta} (\alpha t + 1)^{-\beta} = -\log(\alpha t + 1) \frac{1}{(\alpha t + 1)^{\beta}},$$

we then have

$$-\frac{\partial^2}{\partial \alpha \partial \beta} l = \sum_{t=t_L}^{t_U} y_t \frac{|t|}{\alpha|t|+1} - \gamma \sum_{t=t_L}^{t_U} \frac{|t|}{(\alpha|t|+1)^{\beta+1}} + \gamma\beta \sum_{t=t_L}^{t_U} \log(\alpha|t|+1) \frac{|t|}{(\alpha|t|+1)^{\beta+1}}.$$

When we take the expected value, the first two terms cancel and

$$I_{\alpha\beta} = E\Big(- \frac{\partial^2}{\partial \alpha \partial \beta} l \Big) = \beta\gamma \sum_{t=t_L}^{t_U} \log(\alpha|t|+1) \frac{|t|}{(\alpha|t|+1)^{\beta+1}}.$$

The divergence is the same as in the case of $I_{\alpha\alpha}$.

Similarly, we can evaluate $I_{\beta\beta}, I_{\alpha\gamma}, I_{\beta\gamma}, I_{\gamma\gamma}$ as

$$I_{\beta\beta} = \gamma \sum_{t=t_L}^{t_U} \frac{(\log(\alpha|t|+1))^2}{(\alpha|t|+1)^{\beta}}, \; I_{\alpha\gamma} = -\beta \sum_{t=t_L}^{t_U} \frac{|t|}{(\alpha|t|+1)^{\beta+1}},$$

$$I_{\beta\gamma} = - \sum_{t=t_L}^{t_U} \frac{\log(\alpha|t|+1)}{(\alpha|t|+1)^{\beta}}, \; I_{\gamma\gamma} = \frac{1}{\gamma} \sum_{t=t_L}^{t_U} \frac{1}{(\alpha|t|+1)^{\beta}}.$$

We can show the following results based on our computation of the Fisher information matrix. Since y_{t_0} is Poisson distributed with mean γ and standard deviation $\sqrt{\gamma}$, y_{t_0}/γ converges to 1 in probability as $\gamma \to \infty$. Furthermore $I_{\alpha\alpha}, I_{\alpha\beta}, I_{\beta\beta}$ are linear in γ and MLE is consistent as $\gamma \to \infty$. For large means, the Poisson distribution is well approximated by a normal distribution after normalization and hence the score functions $\partial l/\partial\alpha, \partial l/\partial\beta, \partial l/\partial\gamma$ are approximately normally distributed as $\gamma \to \infty$. The confidence intervals given in the Results section are based on this approximation.

When γ is fixed and $\max(-t_L, t_U) \to \infty$, the elements of the Fisher information matrix diverge to ∞ if and only if $\beta \leq 1$, or equivalently $\sum_{t=t_L}^{t_U} E(y_t) \to \infty$.

Fisher information matrix for the AR(1) model We present the Fisher information matrix for the AR(1) model, i.e., the model in (5) with $s = 1$. We let $t_0 = 0$ for simplicity and assume that $y_0, \ldots, y_T$ are observed. We replace γ with y_0 and consider the conditional likelihood for α and β given y_0.

The conditional expected values, given y_0, are

$$E(y_1|y_0) = y_0 \left(\frac{1}{\alpha+1}\right)^{\beta},$$
$$E(y_2|y_1) = y_1 \left(\frac{\alpha+1}{2\alpha+1}\right)^{\beta}, E(y_2|y_0) = y_0 \left(\frac{1}{2\alpha+1}\right)^{\beta},$$
$$E(y_t|y_{t-1}) = y_{t-1} \left(\frac{(t-1)\alpha+1}{t\alpha+1}\right)^{\beta}, E(y_t|y_0) = y_0 \left(\frac{1}{t\alpha+1}\right)^{\beta}.$$

The conditional likelihood function is

$$L(\alpha,\beta) = \prod_{t=1}^{T} \frac{\mu_t^{y_t}}{y_t!} e^{-\mu_t}, \quad \mu_t = y_{t-1} \left(\frac{(t-1)\alpha+1}{t\alpha+1}\right)^{\beta}.$$

Then the conditional log-likelihood function $l(\alpha,\beta) = \log L(\alpha,\beta)$ is written as

$$\begin{aligned} l(\alpha,\beta) &= \sum_{t=1}^{T} (y_t \log \mu_t - \mu_t - \log y_t!) \\ &= C + \beta \sum_{t=1}^{T} y_t \log \frac{(t-1)\alpha+1}{t\alpha+1} - \sum_{t=1}^{T} y_{t-1} \left(\frac{(t-1)\alpha+1}{t\alpha+1}\right)^{\beta}, \end{aligned}$$

where C does not depend on α, β. The first and second derivatives with respect to α are evaluated as

$$\begin{aligned} -\frac{\partial}{\partial\alpha} l(\alpha,\beta) &= -\beta \sum_{t=1}^{T} y_t \left\{\frac{t-1}{(t-1)\alpha+1} - \frac{t}{t\alpha+1}\right\} - \beta \sum_{t=1}^{T} y_{t-1} \frac{((t-1)\alpha+1)^{\beta-1}}{(t\alpha+1)^{\beta+1}}, \\ -\frac{\partial^2}{\partial\alpha^2} l(\alpha,\beta) &= -\beta \sum_{t=1}^{T} y_t \left\{-\frac{(t-1)^2}{((t-1)\alpha+1)^2} + \frac{t^2}{(t\alpha+1)^2}\right\} \\ &\quad - \beta \sum_{t=1}^{T} y_{t-1} \frac{((t-1)\alpha+1)^{\beta-2}}{(t\alpha+1)^{\beta+2}} \times (-2\alpha t^2 - 2(1-\alpha)t + 1 - \beta). \end{aligned}$$

Taking the expected value, we have

$$I_{\alpha\alpha} = \beta^2 y_0 \sum_{t=1}^{T} \frac{((t-1)\alpha+1)^{-2}}{(t\alpha+1)^{\beta+2}}.$$

The mixed derivative with respect to α and β and its expected value are evaluated as

$$\begin{aligned} -\frac{\partial^2}{\partial\beta\partial\alpha} l(\alpha,\beta) &= -\sum_{t=1}^{T} y_t \left\{\frac{t-1}{(t-1)\alpha+1} - \frac{t}{t\alpha+1}\right\} \\ &\quad - \sum_{t=1}^{T} y_{t-1} \frac{((t-1)\alpha+1)^{\beta-1}}{(t\alpha+1)^{\beta+1}} \times \left\{1 + \beta \log \frac{(t-1)\alpha+1}{t\alpha+1}\right\}, \end{aligned}$$

$$I_{\alpha\beta} = -\beta y_0 \sum_{t=1}^{T} \frac{((t-1)\alpha+1)^{-1}}{(t\alpha+1)^{\beta+1}} \log \frac{(t-1)\alpha+1}{t\alpha+1}.$$

Similarly, the second derivative with respect to β and its expected values are evaluated as

$$I_{\beta\beta} = y_0 \sum_{t=1}^{T} \frac{1}{(t\alpha + 1)^{\beta}} \left(\log \frac{(t-1)\alpha + 1}{t\alpha + 1} \right)^2 .$$

Note that the elements of the Fisher information matrix are proportional to y_0. Also, the series diverges if and only if $\beta \leq 1$. This is the same as in the power-law growth and decay model without autocorrelation.

For the AR(2) models, the evaluation of the Fisher information matrix is difficult, mainly because we cannot separate y_{t-1} and y_{t-2} in $\log \mu_t$.
